# ERP-based evidence for the independent processing of structural and functional action semantics

**DOI:** 10.3389/fnins.2025.1571972

**Published:** 2025-03-26

**Authors:** Yanglan Yu, Qin Huang, Xudong Liu, Shiying Gao, Xuechen Mao, Anmin Li

**Affiliations:** ^1^School of Psychology, Shanghai University of Sport, Shanghai, China; ^2^Department of Physical Education, Nanjing University of Chinese Medicine, Nanjing, China

**Keywords:** manipulative actions, structural actions, functional actions, action semantic processing, cluster-based permutation analysis, event-related potentials (ERPs)

## Abstract

In this study, the semantic processing and neural mechanisms of manipulative actions, categorized as structural actions and functional actions, were examined to assess whether these action types involve independent cognitive processes. Using a cue-stimulus paradigm with event-related potentials (ERPs), we analyzed neural responses to various manipulative actions. Manipulating the semantic congruency of structural actions (congruent vs. incongruent) and functional action types (wave vs. press) revealed distinct neural patterns. We observed distinct neural differences for functional actions in the 30–44 ms, 144–194 ms, 218–232 ms, 300–400 ms, and 562–576 ms windows. Early activation occurred in the left medial superior frontal gyrus, whereas sustained activity spread from the occipital and parietal regions to frontal regions between 144–194 ms and 300–400 ms. Late activation, occurring in the 562–576 ms window, was localized to the left middle frontal gyrus, right orbital inferior frontal gyrus, and right superior occipital gyrus. For structural actions, neural differences emerged in the 456–470 ms and 610–660 ms windows, which activated the parietal and temporal regions, including the left postcentral gyrus and right middle temporal gyrus. These findings suggest that the semantic processing of structural actions is partially independent of functional action cognition at the neural level.

## 1 Introduction

Manipulable objects are defined as those that can be used to achieve specific functional purposes. Primates, including humans, can manipulate these objects, skillfully performing grasping actions and using actions in daily life to meet specific goals (Barsalou, 2008; Tucker and Ellis, 1998). Grasping actions involve physically handling an object on the basis of its external structure, and using actions involve employing the object’s function to achieve a particular purpose (Brandi et al., 2014; Bub et al., 2015). Buxbaum’s two-action systems (2AS) model proposes that the bilateral dorso-dorsal visual pathway forms the structural action system, through which objects are manipulated on the basis of spatial information (structure-based actions, such as grasping and moving objects). In contrast, the left lateral dorso-ventral visual pathway constitutes the functional action system, which extracts the core features of an object used to perform function-based actions, such as the object’s intended purpose (Binkofski and Buxbaum, 2013; Buxbaum and Kalenine, 2010). For example, a hammer’s structural action involves a power grip due to its shape and weight, while its functional action is swinging to drive a nail. In contrast, a pencil requires a precision grip for structural action, and its functional action involves pressing it against paper to write. Scholars suggest that the recognition and cognitive representation of these actions differ: structural actions are processed through online processing, requiring minimal working memory but exhibiting short retention. In contrast, functional actions involve offline processing, requiring more working memory and allowing for longer retention. Thus, the recognition and processing methods for these two actions are considered independent.

Previous studies have shown that behavioral performance differs when the ability to perform the two types of manipulative actions is impaired. Specifically, when the ability to perform structural actions is impaired, subjects are unable to accurately position their hand to reach for and stably grasp an object. This phenomenon is referred to as optic ataxia (Andersen et al., 2014). In contrast, when the ability to perform functional actions is impaired, individuals fail to use familiar objects correctly, which is referred to as apraxia (Goldenberg, 2014). However, importantly, impairment in one type of action ability does not always coincide with impairment in the other. For example, some apraxia patients cannot recognize or perform the correct gestures to use an object, but they can accurately reach for and grasp the object within their visual field (Angela et al., 1995; Jax et al., 2006). Moreover, apraxia patients show greater recognition accuracy for objects that require more complex structural manipulation than for those requiring less complex manipulation (Barde et al., 2007). Additionally, these patients find it more difficult to perform the correct action when visual information is temporarily deprived (Jax et al., 2006). These behavioral manifestations suggest that structural and functional manipulative actions may involve independent systems, both of which rely on visual processing pathways, which supports the 2AS model.

Moreover, current theories have defined specific brain regions involved in spatial activation for processing each type of manipulative action. Recognition of structural actions relies on the dorso-dorsal stream, which passes through visual area V6 and the superior parietal lobule (SPL), ultimately reaching the dorsal premotor area. In contrast, recognition of functional actions relies more heavily on the dorso-ventral stream, which passes through the V5/MT regions and the inferior parietal lobule (IPL), ultimately reaching the ventral premotor area (Augurelle et al., 2003). Further physiological studies on patients with brain injury have shown that optic ataxia is associated primarily with damage to the SPL, intraparietal sulcus (IPS), and parieto-occipital junction (POJ) (Karnath and Perenin, 2005; Perenin and Vighetto, 1988), whereas apraxia is associated with damage to the left IPL (Rueschemeyer et al., 2010; Salazar-López et al., 2016). Additionally, studies comparing brain activation during tasks involving structural and functional manipulation of objects have revealed that judging functional actions activates the left IPL, left inferior frontal gyrus (IFG), and posterior superior temporal gyrus (pSTG) more significantly than judging structural actions does (Buxbaum et al., 2006). Notably, activation in these regions, including the left IPL, left postcentral gyrus, left inferior precentral gyrus, and presupplementary motor area (pre-SMA), exhibits substantial left hemispheric lateralization when viewing objects with a focus on functional manipulation. Although the same brain regions are activated when objects are recognized with a focus on structural manipulation, no lateralization occurs.

However, a study that required participants to judge objects by observing different manipulative actions involving force application revealed that when functional action recognition occurs first, the response time for structural action recognition is significantly delayed (Jax and Buxbaum, 2010, 2012). This finding suggests that recognition of these two types of manipulative actions may not follow entirely independent processing pathways, as previously believed. Instead, some activated brain regions or temporal processing patterns may overlap, leading to reciprocal influences on the recognition response times of both actions, especially when both action recognition tasks are performed sequentially in a limited time frame. This overlap may result in increased depletion of cognitive resources. Embodied cognition theory suggests that cognitive processes are ‘modal simulations,’ where action cognition is closely linked to sensory perception (Barsalou, 2008). Action language activates motor-related brain areas (Courson and Tremblay, 2020), such as hand-related regions when presenting an image of a hammer or foot-related areas when showing a foot stepping on an object (Klepp et al., 2014). This cognitive processing is enhanced by an object’s manipulability, which boosts action recognition (Beauprez et al., 2020; Madan, 2014). Additionally, the neural network activated by action language is similar to that involved in action observation and imagination, engaging regions like Broca’s area, the premotor cortex, the somatosensory cortex, and the posterior middle temporal gyrus (Courson and Tremblay, 2020; Giacobbe et al., 2022). Notably, the posterior temporal-occipital region and prefrontal cortex overlap with areas involved in action observation and imagination, suggesting a similar process in manipulative action cognition. The parietal region plays a key role in processing both types of manipulative actions. Both manipulative actions recognition pathways likely start in the visual cortex (occipito-temporal lobe), pass through the parietal lobe, and reach the motor cortex in the frontal lobe. However, activation in the fronto-parietal region varies slightly depending on the action type: functional actions maybe activate the medial frontal cortex, while structural actions activate the lateral regions.

Furthermore, a functional magnetic resonance imaging (fMRI) study using a priming paradigm demonstrated that performing the correct grasping gesture is necessary for executing functional actions. When a left or right auditory cue was provided, followed by an image of the object grasped on the left or right side, the results indicated that even lateral differences can affect judgments of object manipulation (Knights et al., 2021). However, prior studies have not strictly controlled for the types of manipulative actions, and it remains unclear whether differences in the types of structural actions significantly influence the recognition of functional actions. Structural actions can be classified into power grasping and precision grasping based on the shape and weight of the object (Bergstrom et al., 2021); functional actions also vary according to purpose and intent (Lycett, 2013). Although the impact of semantic congruency on action recognition is known (Liu et al., 2022; Monaco et al., 2023), whether the semantic congruency of structural action types influences the recognition of functional actions has not yet been tested. Additionally, the temporal differences in information processing and action recognition for these two types of actions remain unclear. Research on embodied cognition suggests that brain regions activated by action language, observation, and imagination overlap significantly (Giacobbe et al., 2022). This study will focus on operational action language by categorizing two action types (structural: pinch/clench; functional: wave/press) to select manipulable objects. We will manipulate the semantic consistency of structural actions in cue and target stimuli to explore whether this affects the cognitive processing of functional actions, particularly at the neural level.

Therefore, in this study, we proposed a factorial design combined with a cue–target paradigm to select manipulable objects as experimental stimuli according to their appearance and action types. This design varies the semantic congruency (congruent, incongruent) of structural actions and the action types (wave, press) of functional actions. We aimed to analyze whether semantic processing of structural action information affects the recognition of functional actions, thus investigating the independence of processing these two types of actions from a new perspective. At the neurophysiological level, we utilized electroencephalography (EEG) to directly observe the temporal sequence of neural activity corresponding to each action and identify the brain regions involved in these processes. We predict that, behaviorally, semantic consistency will speed up judgments of functional actions, while at the neural level, the effects of structural actions semantic congruency and functional actions recognition will occur in distinct time windows and activate different brain regions. The effect of structural actions is achieved through manipulating semantic coherence, while the effect of functional actions is achieved through objects of different action types.

## 2 Methods

### 2.1 Participants

Thirty students from Shanghai University of Sport participated in this experiment (14 males and 16 females, aged 20–24 years, mean ± SD = 20.10 ± 1.8 years). All participants had normal or corrected vision, had no significant differences in body mass index (BMI), were right-handed, were healthy, were free from neurological or muscular diseases, and had not recently taken psychoactive medications. The experimental requirements and procedures were explained beforehand, and written informed consent was obtained. The participants were compensated on the basis of their participation time. In this study, participants provided informed written consent and were paid for their participation. The study followed ethical guidelines set forth by the Declaration of Helsinki and was approved by the local ethics committee at Shanghai University of Sport in China.

### 2.2 Stimuli

The target object stimulus images were selected from the Bank of Standardized Stimuli (BOSS) (Mathieu et al., 2014). We categorized manipulable objects according to two grasping dimensions (pinch and clench) and two using dimensions (wave and press) (Buxbaum et al., 2006), resulting in four manipulative action combinations: pinch and wave, pinch and press, clench and wave, and clench and press. Taking the hammer as an example, its large mass requires a power grip for structural actions, while the functional actions involve waving it to drive a tack (Fu et al., 2018). Thus, its manipulative action combination is clench and wave. In contrast, the nail clipper’s small size and light weight require a pinch grip for structural actions, with the functional actions involving pressing to cut nails. Therefore, its manipulative action combination is pinch and press.

**Figure 1:**
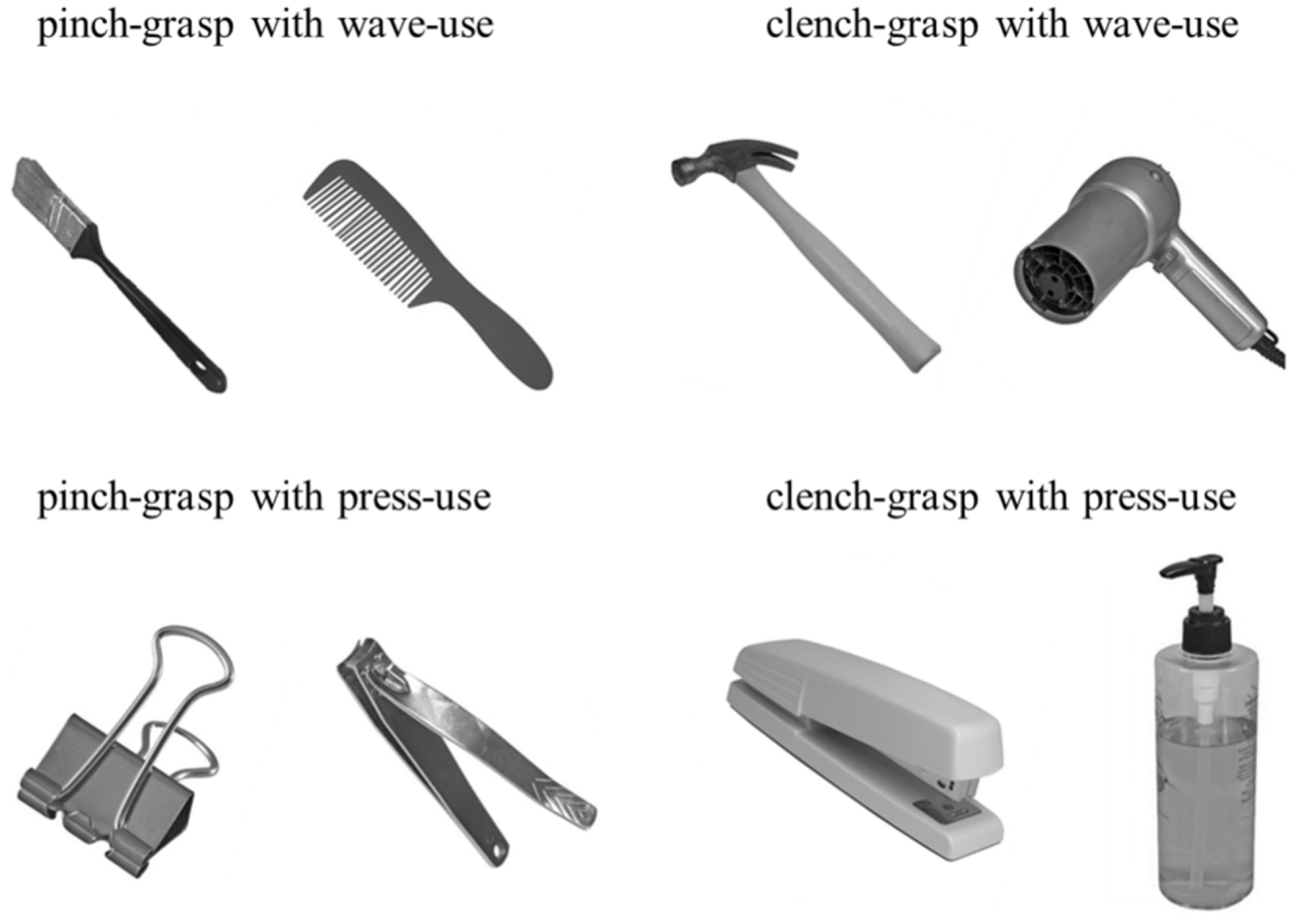
Manipulable object stimuli. Using a chi-square test for independence, we identified 8 objects with highly consistent action selections categorized into four action types: pinch and wave, pinch and press, clench and wave, and clench and press, with each combination featuring two distinct objects.

To ensure consistent participant responses, we recruited 198 individuals (aged 18–25 years) to classify grasping and using actions for the selected objects before the experiment. We verified significant agreement in their selections across the four action combinations via the chi-square test for independence (Table 1). A total of 8 objects (two per combination) were used as target stimuli (Figure 1). The stimuli were grayscale adjusted and displayed on a calibrated screen (1,024 × 768 pixels, 60 Hz refresh rate) 45 cm from the participant’s eyes. The presentation was controlled using Psychtoolbox in MATLAB (Brainard, 1997; Pelli, 1997). The objects were shown at a consistent angle with handles tilted left by 45°, subtending a visual angle of 3.8°. Responses were collected via a keyboard, and event-related potential (ERP) analysis was performed to explore temporal brain activation dynamics.

**Table 1:** Chi-square tests for the selection of manipulative actions for eight objects.

Manipulable object	Structural actions (*N* = 198)		Functional actions (*N* = 198)		Chi-square test (Chi-Square value, *p-*value)	
	Pinch	Clench	Wave	Press	Structural actions	Functional actions
Brush	195	3	198	0	186.18, <0.001	198, <0.001
Comb	193	5	196	2	178.92, <0.001	190.94, <0.001
Hammer	0	198	195	3	198, <0.001	186.18, <0.001
Dryer	2	196	192	6	190.94, <0.001	174.72, <0.001
Clamp	196	2	0	198	190.94, <0.001	198, <0.001
Scissor	198	0	0	198	198, <0.001	198, <0.001
Stapler	2	196	2	196	190.94, <0.001	190.94, <0.001
Bottle	3	195	1	197	186.18, <0.001	194.14, <0.001

### 2.3 Task and procedure

The experiment consisted of two phases: an action testing phase and a main experimental phase. The testing phase (20 min) assessed participants’ familiarity with the two types of manipulative actions and ensured the correct application of action combinations in the main experiment. The main experimental phase lasted approximately 60 min. In each trial, the participants selected the appropriate action for a presented object via a keypress.

**Figure 2:**
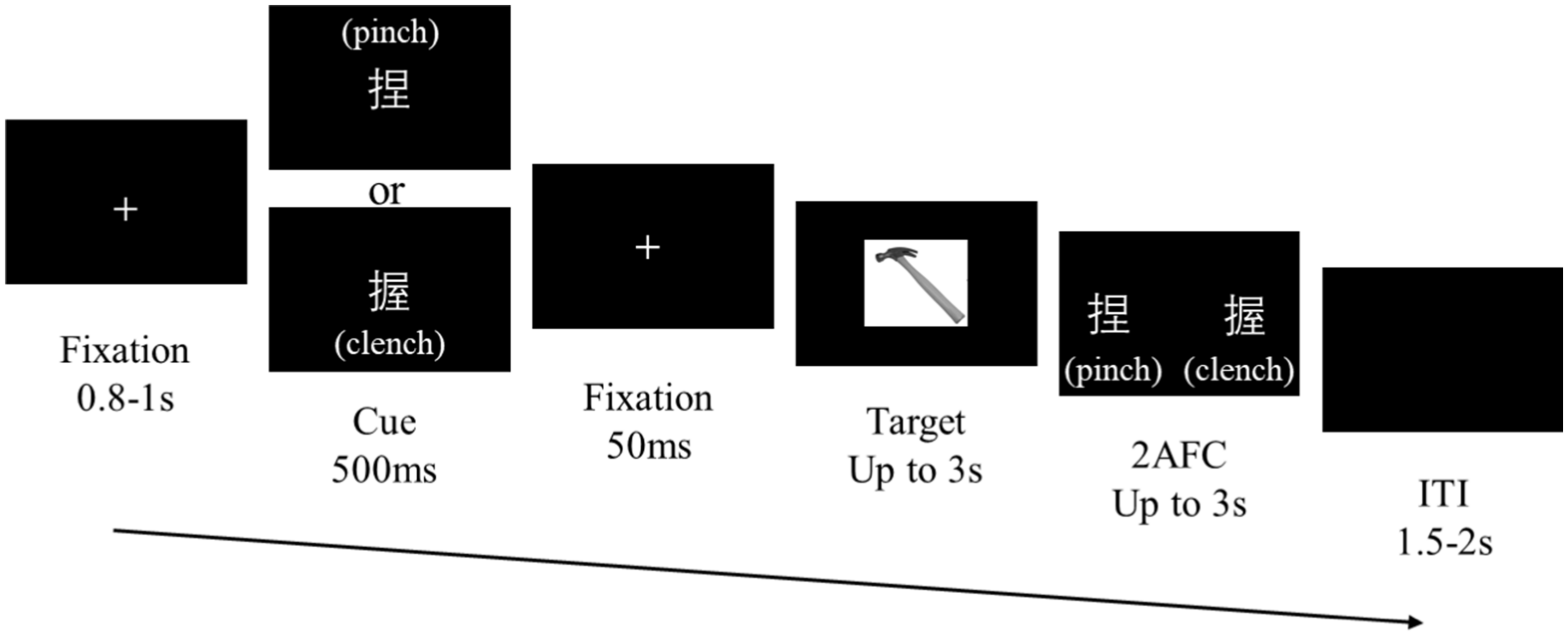
Procedure design. Task procedure. Cue stimuli are randomly displayed as either ‘捏’ (pinch) or ‘握’ (clench). Fifty percent of the trials featured congruent structural actions between the cue and target, and the rest of the trials were incongruent.

Each trial began with a white ‘+’ fixation point at the center of the screen (Figure 2) for 0.8–1 s, which was randomly timed to minimize expectancy effects. Next, a structural action cue, presented as the Chinese character meaning pinch (‘捏’) or clench (‘握’), appeared for 500 ms, followed by a 50 ms fixation. An image of a manipulable object was subsequently presented as the target stimulus. The participants responded as quickly and accurately as possible using the ‘ ↑ ’ or ‘ ↓ ’ keys to identify the functional action associated with the object (‘wave’ or ‘press’). The target stimulus disappeared after a response or after 3 s with no response.

After the target task, a forced-choice screen appeared to test recognition of the preceding cue. Two words, ‘捏’ (pinch) and ‘握’ (clench), were displayed in random horizontal positions, and the participants used the ‘←’ or ‘→’ keys to indicate their choice according to the character. The forced-choice answers alternated left and right screen positions across trials to prevent biases, and the characters appeared at locations that did not overlap with the object image to prevent masking effects. The intertrial intervals featured a black screen lasting 1.5–2 s.

The experiment had a 2 (semantic congruent/semantic incongruent) × 2 (wave/press) factorial design, manipulating the semantic information of structural actions and the types of functional actions. Four blocks of 128 trials each were conducted, with 50% of the trials showing a congruent cue and target structural action and an equal distribution of target object types (1:1 ratio).

### 2.4 EEG data acquisition

The EEG data were recorded using the Brain Vision Recorder 2.0 system (Brain Products Company, Germany), with 64 electrodes positioned according to the 10–20 system. The FCz electrode served as the reference electrode, and the AFz electrode served as the ground. Vertical electrooculogram (VEOG) signals were collected for offline artifact correction. The signals were amplified with a 0.01–100 Hz bandpass filter and digitized at 500 Hz using a BrainAmp amplifier, and the electrodes were maintained at an impedance less than 5 kΩ.

### 2.5 EEG data analysis

The EEG data were analyzed using the EEGLAB toolbox in MATLAB (Delorme and Makeig, 2004; Iversen and Makeig, 2014). Independent component analysis has been shown to reduce EOG artifacts (Gratton et al., 1983). Data were segmented from 200 ms before the cue to 2, 000 ms after the target presentation, and trials with muscle artifacts or voltages exceeding ±80 μV were excluded. The data were low-pass filtered at 30 Hz, with baseline correction applied to the 200 ms window before cue onset.

To investigate the neural associations between structural and functional actions, we employed a cluster-based permutation test (Maris et al., 2007; Yu et al., 2019). This approach, which does not require predefined time windows or regions, is effective for identifying differences across sensors and time samples while controlling for multiple comparisons. However, the sensitivity of the approach is limited for prolonged and spatially extensive activations. To enhance analytical precision, we preselected time windows of interest on the basis of regions with strong and potentially differentiable brain activity.

**Figure 3:**
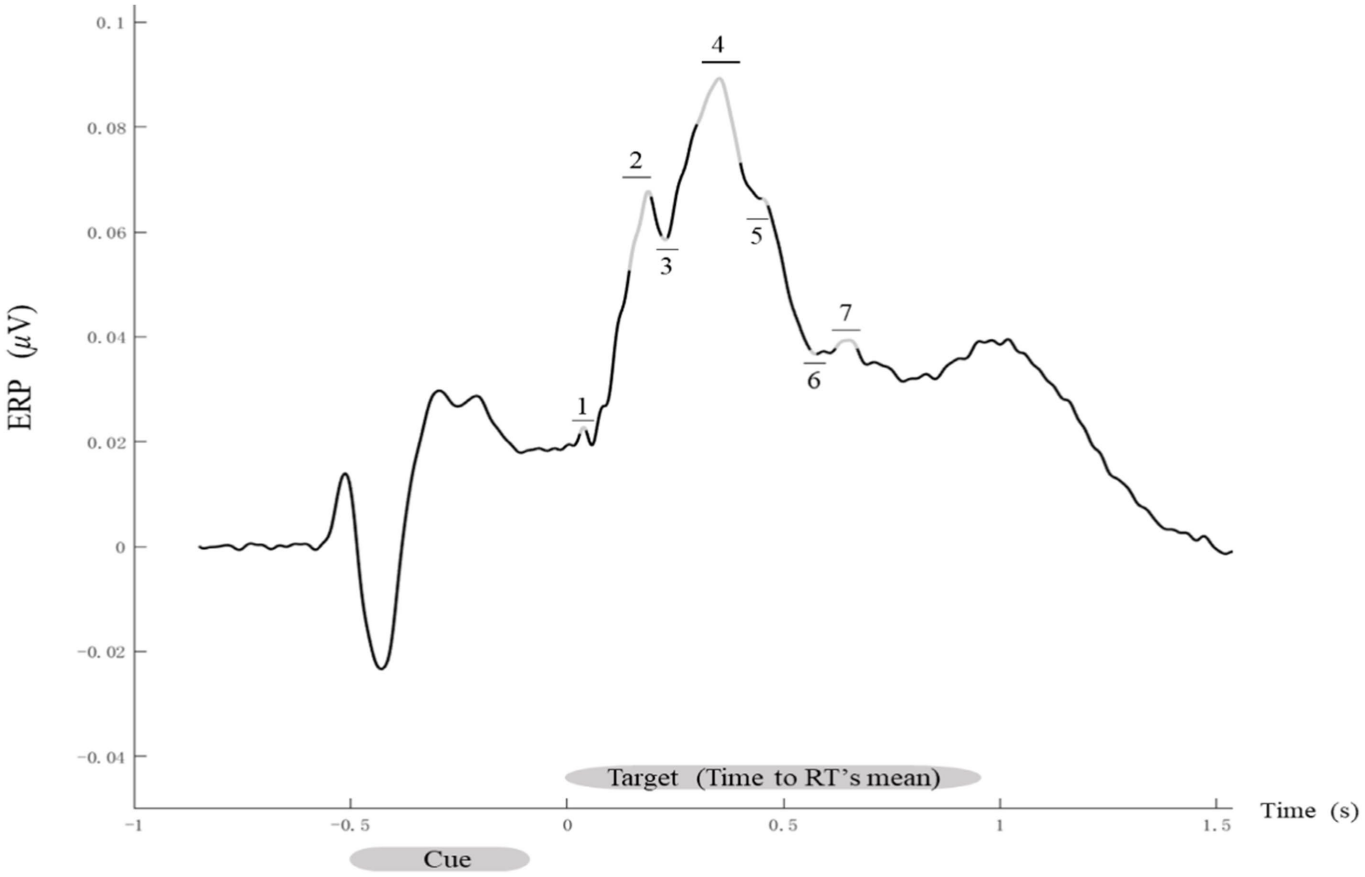
Time course of evoked ERP signals. Grand-average ERP waveforms. The ERP signals evoked by all conditions for each participant were averaged, with data analysis focusing on the seven time windows marked by gray segments with numbered labels: 1 (30–44 ms), 2 (144–194 ms), 3 (218–232 ms), 4 (300–400 ms), 5 (456–470 ms), 6 (562–576 ms), and 7 (610–660 ms).

The time windows were identified by calculating the average ERPs for each participant, electrode, and experimental condition. On the basis of grand-average waveforms across all the participants and conditions, the following seven time windows were defined: 30–44, 144–194, 218–232, 300–400, 456–470, 562–576, and 610–660 ms after target stimulus onset (Figure 3).

A repeated-measures analysis based on a two-tailed cluster permutation test, as proposed by Maris et al. (2007) and Oostenveld et al. (2011) and implemented in the FieldTrip toolbox, was employed to investigate the neural correlates of structural action semantics and functional action types during multiple comparisons (sensors, time samples). Specifically, a repeated-measures *t*-test was conducted on each signal sample (sensor, time point) from correct trials in the action judgment task. The analysis focused on structural action semantics (congruent/incongruent) and functional action types (wave/press). For each main effect, samples were clustered according to temporal and spatial adjacency, with sensors considered adjacent at a distance of less than 4 cm. Also, we replaced the sample data 2,000 times and set 0.025 as the cluster threshold. Samples with positive and negative t values were clustered separately for bilateral testing.

Each cluster defined by spatial and temporal processes was assigned a value equal to the sum of the *t* values of all samples within that cluster. To assess statistical significance, the original event-related data were randomly permuted for each participant and condition. The clustering process was then applied to the randomized data to measure the maximum cluster t value in each region of interest. After 2,000 randomizations, the distribution of the maximum cluster *t* values under the null hypothesis was estimated. If the original statistic exceeded the 97.5% threshold of the randomized data, controlling for multiple comparisons using the maximum statistic, the null hypothesis was rejected (*p* < 0.05).

### 2.6 Source localization

Cortical current density maps were derived using a distributed model with 15,000 dipoles. These dipoles were loosely mapped onto the cortical mantle of a standard brain model using BrainVISA software (Montreal Neurological Institute).^1^ Source localization and surface visualization were conducted with Brainstorm (Tadel et al., 2011), a free, publicly available tool under the GNU General Public License.^2^ Cortical current maps were computed from EEG time series using the weighted minimum-norm estimation (wMNE) for each participant and condition (congruent wave, congruent press, incongruent wave, and incongruent press). These cortical currents were then averaged across participants and the seven time windows of interest (30–44, 144–194, 218–232, 300–400, 456–470, 562–576, and 610–660 ms).

Source values related to structural action semantics were obtained via t tests comparing semantic-congruent (average of semantic-congruent wave and press conditions) and semantic-incongruent (average of semantic-incongruent wave and press conditions) conditions. A similar procedure was used for calculating source values linked to functional action types. Activated sources were defined as clusters of at least 12 contiguous voxels with *t* values exceeding 1.75, corresponding to a *p*-value of 0.05 (uncorrected for multiple comparisons).

## 3 Results

### 3.1 Behavior

#### 3.1.1 Subjective measurement of structural action semantic information

The participants accurately identified the structural action cue stimulus in the majority of the trials, with a hit rate (mean ± standard error of the mean [SEM] = 96.83 ± 0.66%) significantly exceeding the false alarm rate (mean ± SEM = 2.69 ± 0.53%) (paired *t*-test, *p* < 0.001). Moreover, the discrimination index (d’) was significantly greater than zero [paired *t*-test, *t*(29) = 28.23, *p* < 0.001], and the likelihood ratio (*β*) significantly deviated from 1 [paired *t*-test, *t*(29) = −4.04, *p* < 0.001]. These findings confirm that participants reliably recognized the structural action cue stimulus. Subsequent analyses focused on trials where the cue stimuli were correctly identified.

#### 3.1.2 Priming effect of structural action semantics

Structural action semantic information was divided into two categories—congruent and incongruent—according to the alignment between cue and target stimuli. If the recognitions of structural and functional actions are not processed independently, the congruency of structural action information may influence participants’ performance in functional action identification tasks. To minimize the impact of outliers on reaction times (RTs), trials with RTs less than 200 ms or greater than 1,500 ms were excluded. Additionally, data exceeding two standard deviations from the mean were identified and removed programmatically.

A 2 × 2 repeated-measures analysis of variance (ANOVA) was performed to examine the effects of structural action semantic congruency (congruent vs. incongruent) and functional action type (wave vs. press) on response accuracy and RT during functional action judgments.

**Figure 4:**
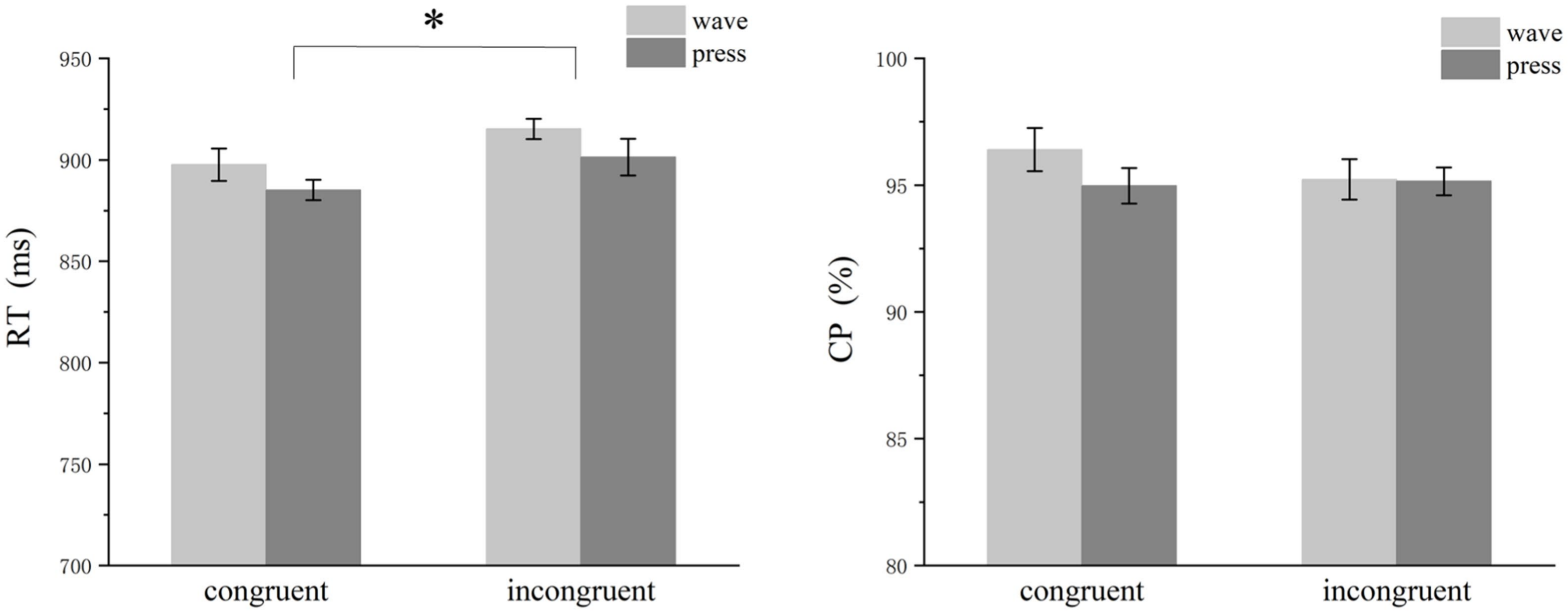
Bar graph of reaction times and accuracy. The participants responded significantly faster under congruent conditions than under incongruent conditions. Additionally, a significant difference was detected for different functional action types. However, semantic congruency did not significantly affect response accuracy.

The analysis revealed a significant effect of semantic congruency on RT. The participants responded faster under congruent conditions than under incongruent conditions [congruent: mean ± SEM = 892.72 ± 6.46 ms; incongruent: mean ± SEM = 908.53 ± 7.24 ms; *F*(1,29) = 11.633, *p* = 0.002, η^2^p = 0.279]. However, semantic congruency did not significantly affect response accuracy [congruent: mean ± SEM = 95.69 ± 0.62%; incongruent: mean ± SEM = 95.49 ± 0.51%; *F*(1,29) = 0.316, *p* = 0.578, η^2^p = 0.012] (Figure 4). Additionally, a significant main effect of functional action type was observed: RTs for the press action were significantly faster than those for the wave action [press: mean ± SEM = 893.07 ± 7.49 ms; wave: mean ± SEM = 917.15 ± 6.39 ms; *F*(1,29) = 8.106, *p* = 0.008, η^2^p = 0.213].

### 3.2 Electrophysiological correlations of the two manipulative actions

Considering that Cluster-based permutation tests identify broad spatiotemporal clusters spanning multiple time points and channels, we conducted an ANOVA on the average activation amplitudes within the window of interest. This allowed for a more precise examination of ERP differences across conditions within these time windows.

#### 3.2.1 Time window 30–44 ms

Cluster-based permutation tests revealed significant differences between the different functional action types within the 30–44 ms time window after the target stimulus presentation (*p* < 0.05). Specifically, one positive cluster and one negative cluster were detected in the spatiotemporal domain, with the positive cluster showing significant differences (*p* = 0.0190).

**Figure 5:**
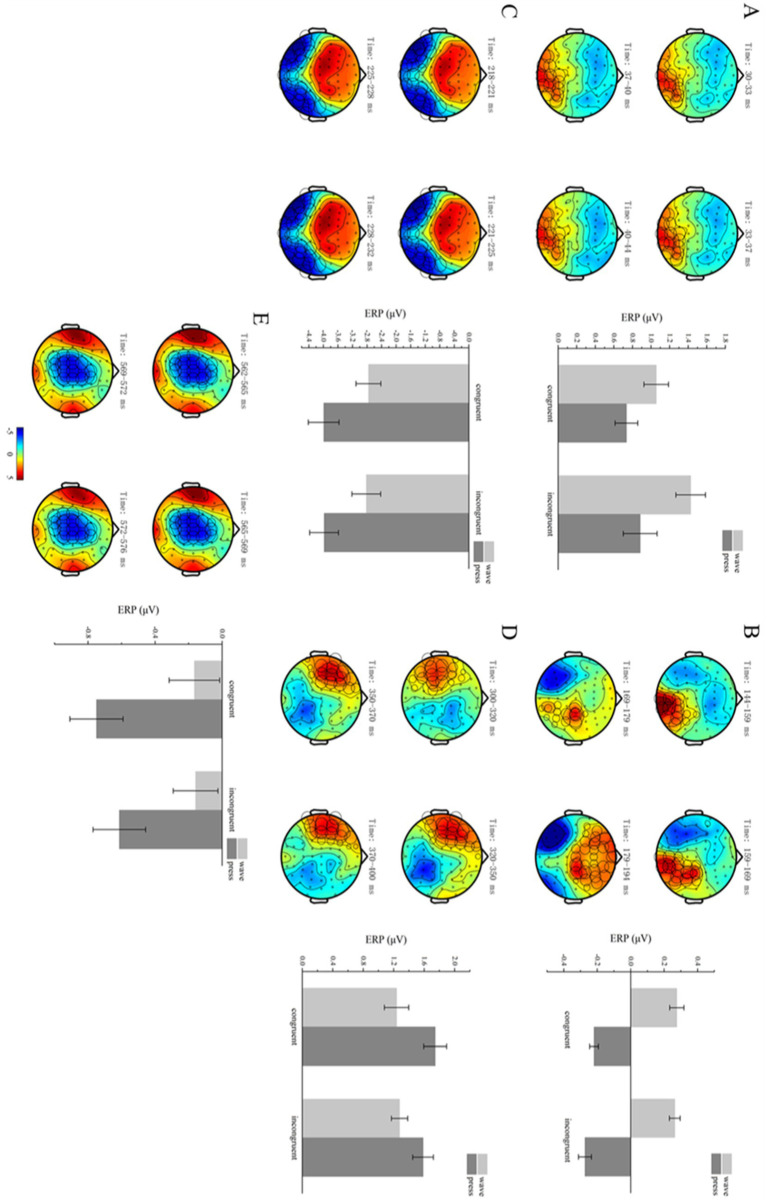
Functional action-related clusters. **(A–E)** Functional action-related effects occurred in the time windows of 30–44, 144–194, 218–232, 300–400, and 562–576 ms. On the left, brain topographies show significant clusters, with color differences representing the activation amplitude between wave and press trials. The cluster locations are indicated by circles, with the diameter proportional to the extent of activation. On the right, bar graphs show average ERP activations for significant positive **(A,B,D)** and negative **(C,E)** clusters, with error bars representing standard error.

The mean ERP activation was computed for each condition and participant within the positive cluster. A 2 × 2 repeated-measures ANOVA was conducted on the electrode signals across the four conditions: structural action semantic congruency (congruent vs. incongruent) and functional action type (wave vs. press). The results revealed a significant main effect of functional action type [*F*(1,29) = 10.417, *p* = 0.030, η^2^p = 0.264]. However, no significant main effect of semantic congruency [*F*(1,29) = 1.926, *p* = 0.176] or significant interaction effect between the two factors [*F*(1,29) = 2.057, *p* = 0.153] was observed (Figure 5A) (collated *p*-value of ANOVA see Table 2).

**Table 2:** ANOVA results of mean ERP activations in time windows.

Time window	Structural action semantic congruency × Functional action type ANOVA result *p*-value		
	Functional action type Main effect	Structural action semantic congruency Main effect	Interaction effect
30–44 ms	**0.030**	0.176	0.153
144–194 ms	**<0.001**	0.338	0.603
218–232 ms	**<0.001**	0.732	0.670
300–400 ms	**<0.001**	0.268	0.105
562–576 ms	**<0.001**	0.268	0.402
456–470 ms	0.794	**0.001**	0.241
610–660 ms	0.147	**0.003**	0.808

The bold values represent significant effects with *p*-values < 0.05.

#### 3.2.2 Time window 144–194 ms

Cluster-based permutation tests revealed significant differences between the two functional action types within the 144–194 ms time window after target stimulus presentation (*p* < 0.05). Specifically, one positive cluster and one negative cluster were detected in the spatiotemporal domain, with the positive cluster showing significant differences (*p* = 0.0020).

The mean ERP activation was computed for each condition and participant within the positive cluster. A 2 × 2 repeated-measures ANOVA was conducted on the electrode signals across the four conditions: structural action semantic congruency (congruent vs. incongruent) and functional action type (wave vs. press). The results revealed a significant main effect of functional action type [*F*(1,29) = 86.756, *p* < 0.001, η^2^p = 0.749]. However, no significant main effect of semantic congruency [*F*(1,29) = 0.949, *p* = 0.338] or significant interaction effect between the two factors [*F*(1,29) = 0.277, *p* = 0.603] was observed (Figure 5B).

#### 3.2.3 Time window 218–232 ms

Cluster-based permutation tests revealed significant differences between the two functional action types within the 218–232 ms time window after the target stimulus presentation (*p* < 0.05). Specifically, one negative cluster and one positive cluster were detected in the spatiotemporal domain, with the negative cluster showing significant differences (*p* = 0.0010).

The mean ERP activation was computed for each condition and participant within the positive cluster. A 2 × 2 repeated-measures ANOVA was conducted on the electrode signals across the four conditions: structural action semantic congruency (congruent vs. incongruent) and functional action type (wave vs. press). The results revealed a significant main effect of functional action type [*F*(1,29) = 35.799, *p* < 0.001, η^2^p = 0.552]. However, no significant main effect of semantic congruency [*F*(1,29) = 0.119, *p* = 0.732] or significant interaction effect between the two factors [*F*(1,29) = 0.182, *p* = 0.670] was observed (Figure 5C).

#### 3.2.4 Time window 300–400 ms

Cluster-based permutation tests revealed significant differences between the two functional action types within the 300–400 ms time window after the target stimulus presentation (*p* < 0.05). Specifically, one positive cluster and one negative cluster were detected in the spatiotemporal domain, with the positive cluster showing significant differences (*p* = 0.0020).

The mean ERP activation was computed for each condition and participant within the positive cluster. A 2 × 2 repeated-measures ANOVA was conducted on the electrode signals across the four conditions: structural action semantic congruency (congruent vs. incongruent) and functional action type (wave vs. press). The results revealed a significant main effect of functional action type [*F*(1,29) = 22.204, *p* < 0.001, η^2^p = 0.434]. However, no significant main effect of semantic congruency [*F*(1,29) = 1.277, *p* = 0.268] or significant interaction effect between the two factors [*F*(1,29) = 02.803, *p* = 0.105] was observed (Figure 5D).

#### 3.2.5 Time window 562–576 ms

Cluster-based permutation tests revealed significant differences between the two functional action types within the 562–576 ms time window after the target stimulus presentation (*p* < 0.05). Specifically, one negative and two positive clusters were detected in the spatiotemporal domain, with the negative cluster showing significant differences (*p* = 0.0010).

The mean ERP activation was computed for each condition and participant within the negative cluster. A 2 × 2 repeated-measures ANOVA was conducted on the electrode signals across the four conditions: structural action semantic congruency (congruent vs. incongruent) and functional action type (wave vs. press). The results revealed a significant main effect of functional action type [*F*(1,29) = 24.708, *p* < 0.001, η^2^p = 0.460]. However, no significant main effect of semantic congruency [*F*(1,29) = 1.273, *p* = 0.268] or significant interaction effect between the two factors [*F*(1,29) = 0.723, *p* = 0.402] was observed (Figure 5E).

#### 3.2.6 Time window 456–470 ms

Cluster-based permutation testing revealed significant differences between the structural action semantic congruency conditions (congruent vs. incongruent) within the 456–470 ms time window after the target stimulus presentation (*p* < 0.05). One negative and one positive cluster were identified. Significant differences were found in the negative cluster (*p* = 0.0390).

**Figure 6:**
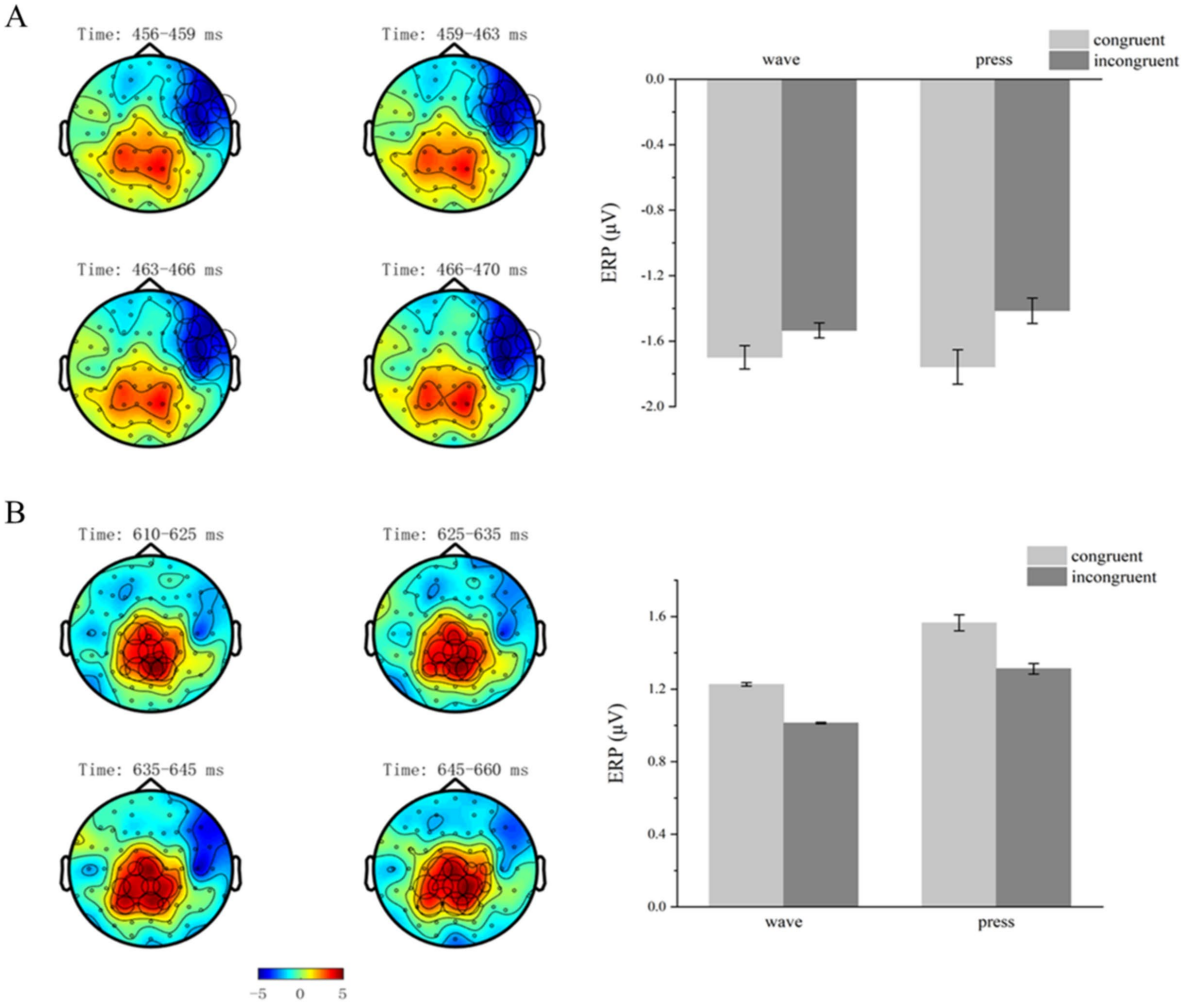
Structural action-related clusters. **(A,B)** Structural action-related effects occurred in the time windows 456–470 and 610–660 ms. On the left, brain topographies show significant clusters, with color differences representing the activation amplitude between semantic-congruent and semantic-incongruent trials. The cluster locations are indicated by circles, with the diameter proportional to the extent of activation. On the right, bar graphs show average ERP activations for significant positive **(B)** and negative **(A)** clusters, with error bars representing standard error.

The mean ERP activation was calculated for each participant and condition within the negative cluster. A 2 × 2 repeated-measures ANOVA was performed on the electrode signals across the four conditions: structural action semantic congruency (congruent vs. incongruent) and functional action type (wave vs. press). The analysis revealed a significant main effect of semantic congruency [*F*(1,29) = 14.087, *p* = 0.001, η^2^p = 0.327]. However, no significant main effect of functional action type [*F*(1,29) = 0.070, *p* = 0.794] or significant interaction effect between the two factors [*F*(1,29) = 1.435, *p* = 0.241] was observed (Figure 6A).

#### 3.2.7 Time window 610–660 ms

Cluster-based permutation testing revealed significant differences between the structural action semantic congruency conditions (congruent vs. incongruent) within the 610–660 ms time window after the target stimulus presentation (*p* < 0.05). One negative and one positive cluster were detected. Significant differences were found in the negative cluster (*p* = 0.0310).

The mean ERP activation was calculated for each participant and condition within this significant cluster. A 2 × 2 repeated-measures ANOVA was performed on the electrode signals across the four conditions: structural action semantic congruency (congruent vs. incongruent) and functional action type (wave vs. press). The analysis revealed a significant main effect of semantic congruency [*F*(1,29) = 10.807, *p* = 0.003, η^2^p = 0.271]. However, no significant main effect of functional action type [*F*(1,29) = 2.286, *p* = 0.147] or significant interaction effect between the two factors [*F*(1,29) = 0.060, *p* = 0.808] was observed (Figure 6B).

The effects of structural action semantic congruency were primarily observed in the 456–470 ms and 610–660 ms time windows after the target stimulus presentation. In contrast, the effects of functional action types appeared earlier, specifically in the 30–44 ms time window, and persisted in four additional windows: 144–194, 218–232, 300–400, and 562–576 ms. These findings suggest temporally independent processing for structural and functional actions, with semantic congruency primarily affecting action processing.

### 3.3 Source localization

To examine the influence of brain regions on the two main effects, we employed a distributed source model. For the EEG cap used in this experiment, following the 10–20 system, the anatomical template provided by Brainstorm was used for head model analysis. We first modeled the neural responses for the functional action types ‘wave’ and ‘press’ separately and then computed the differences across the five time windows identified above: 30–44, 144–194, 218–232, 300–400, and 562–576 ms.

**Figure 7:**
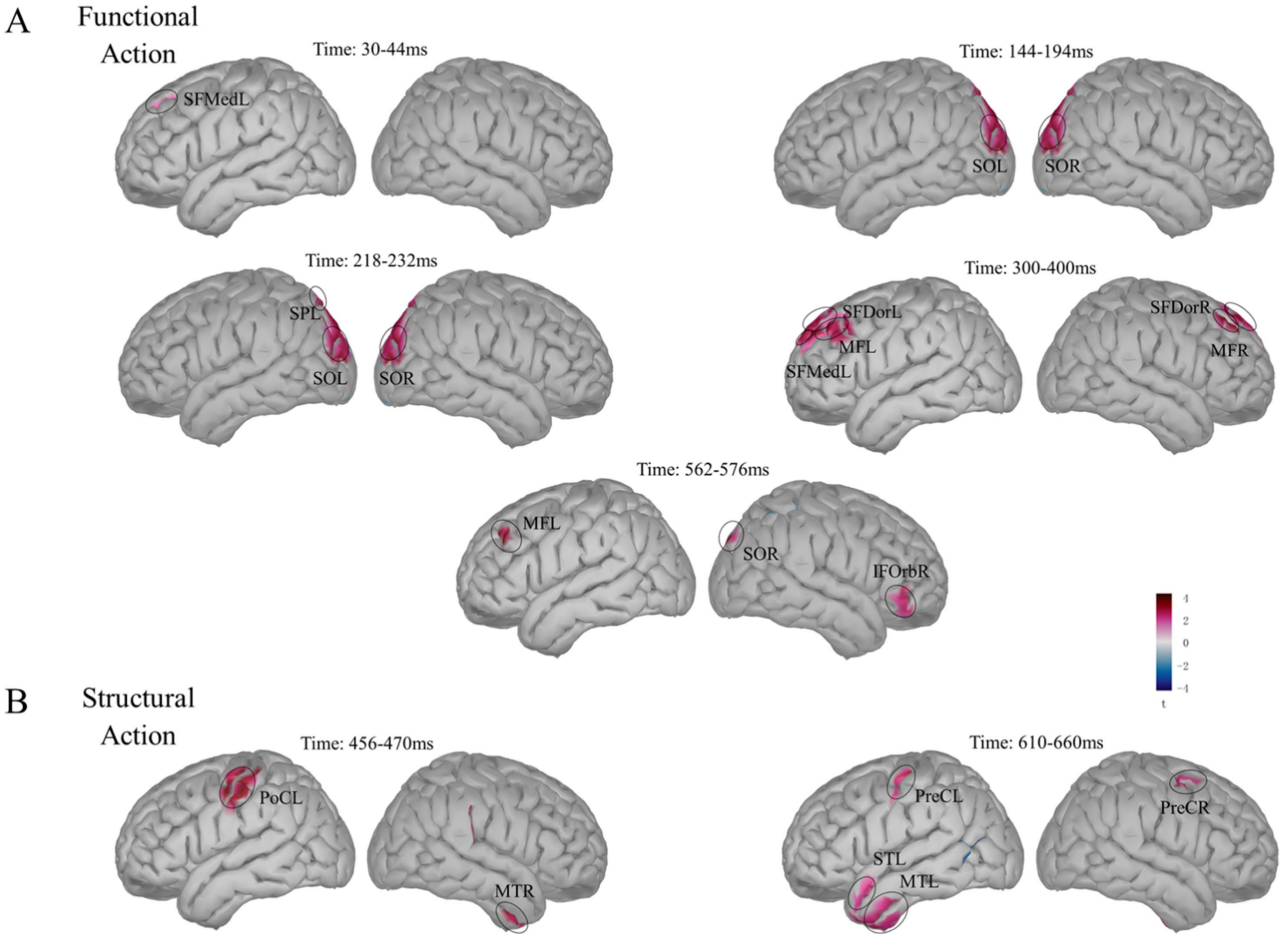
Source localization of the main effects. **(A)** Differences in functional action types. 30–44 ms: left medial superior frontal gyrus (SFMedL). 144–194 ms: left and right superior occipital gyrus (SOL, SOR). 218–232 ms: SOL, SOR and left superior parietal gyrus (SPL). 300–400 ms: left dorsal superior frontal gyrus (SFDorL), SFMedL, left middle frontal gyrus (MFL), right dorsal superior frontal gyrus (SFDorR), and right middle frontal gyrus (MFR). 562–576 ms: MFL, right orbital inferior frontal gyrus (IFOrbR), and SOR. **(B)** Differences in structural action semantic congruency. 456–470 ms: left postcentral gyrus (PoCL) and right middle temporal gyrus (MTR). 610–660 ms: left and right precentral gyrus (PreCL, PreCR), left temporal pole: middle temporal gyrus (TPOmidL), and left temporal pole: superior temporal gyrus (TPOsupL).

In the 30–44 ms window, response differences between the wave and press action types were observed in the left medial superior frontal gyrus (Figure 7A); the coordinates of the activated regions are listed in Table 3. During the 144–194 ms period, significant response differences related to functional action types were found in both the left and right superior occipital gyrus. In the 218–232 ms window, in addition to these regions, response differences between the action types were also detected in the left superior parietal gyrus. During the 300–400 ms window, significant response differences in the wave and press actions were observed in the left dorsal superior frontal gyrus, left medial superior frontal gyrus, left middle frontal gyrus, right dorsal superior frontal gyrus, and right middle frontal gyrus. In the 562–576 ms window, the activation in the frontal–parietal regions gradually decreased, although significant differences related to functional action types remained in the left middle frontal gyrus and right orbital IFG. Additionally, significant activation was found in higher-level visual areas, including the right superior occipital gyrus.

**Table 3:** MNI coordinates (mm) of the activated regions.

	Times	Regions		Coordinates (mm)		
				*x*	*y*	*z*
Functional actions	30–44 ms	SFMedL	Frontal_Sup_Medial_L	−12.5	53.5	42
	144–194 ms	SOL	Occipital_Sup_L	−32.4	−84.3	38.4
		SOR	Occipital_Sup_R	22.4	−85.4	42.8
	218–232 ms	SOL	Occipital_Sup_L	−32.4	−84.3	38.4
		SOR	Occipital_Sup_R	22.4	−85.4	42.8
		SPL	Parital_Sup_L	−39.1	−83.4	30.2
	300–400 ms	SFDorL	Frontal_Sup_Dorsal_L	−24.2	52.6	36.7
		SFMedL	Frontal_Sup_Medial_L	−5.7	27	63.4
		MFL	Frontal_Mid_L	−40.8	26.7	45.7
		SFDorR	Frontal_Sup_Dorsal_R	14.6	46.6	48.7
		MFR	Frontal_Mid_R	33	37.5	46.4
	562–576 ms	MFL	Frontal_Mid_L	−36.9	37.3	35.4
		IFOrbR	Frontal_Inf_Orbital_R	52.8	40.6	−9.1
		SOR	Occipital_Sup_R	8.5	−91.5	29.5
Structural actions	456–470 ms	PoCL	Postcentral_L	−58.9	−20.8	49.3
		MTR	Temporal_Mid_R	52.7	−2.1	−43.1
	610–660 ms	PreCL	Precentral_L	−43.1	−13.7	62.1
		PreCR	Precentral_R	42.4	−1.6	56.3
		TPOmidL	Temporal_Pole_Mid_L	−45.4	−2.2	−49.5
		TPOsupL	Temporal_Pole_Sup_L	−49.8	12.9	−29.1

Using the same approach, we modeled the neural responses for structural action information congruency (congruent vs. incongruent) and calculated the differences in the 456–470 and 610–660 ms windows. In the 456–470 ms window, significant response differences in structural action semantic congruency were observed in the left postcentral gyrus and right middle temporal gyrus (Figure 7B). In the 610–660 ms window, the differences were concentrated in the precentral gyrus of both hemispheres, with increased activation in the temporal lobe, particularly in higher cognitive regions, including the middle temporal gyrus and superior temporal gyrus of the left temporal pole.

## 4 Discussion

In this study, the influence of the semantic processing of structural actions on the recognition of functional actions was investigated. We differentiated the two manipulative actions according to their corresponding object–action associations. Behaviorally, we found that structural action semantic congruency significantly reduced RTs for functional action judgments, with a notable main effect of functional action type. Cluster-based permutation tests revealed distinct neural mechanisms for processing the two types of actions. Structural action semantics-related activations occurred between 456–470 and 610–660 ms after target stimulus presentation, whereas differences in functional action types emerged earlier, at 30–44 ms, 144–194 ms, 218–232 ms, 300–400 ms, and at 562–576 ms. These findings suggest temporal differences in the cognitive processing of the two manipulative actions.

### 4.1 Functional action type-related effects

We manipulated stimulus variables using physically identical stimuli and observed neural responses linked to functional actions across five time windows (30–44, 144–194, 218–232, 300–400, and 562–576 ms). Notably, differences related to functional actions emerged as early as the 30–44 ms window and persisted throughout the subsequent time intervals. These findings support those of previous studies on early visual action processing differences (Catalano et al., 2021; Chen et al., 2017; Conty et al., 2007; Roeber et al., 2008; Wyart et al., 2011). Additionally, significant activation was observed in the left medial superior frontal gyrus during this early period, whereas activation in the bilateral superior occipital gyrus and left superior parietal gyrus was noted during the 218–232 ms window. These results reinforce prior research indicating that brain regions associated with functional action recognition are located primarily in the frontal–parietal and occipital cortices (Kleineberg et al., 2022; Moguilner et al., 2021; Velji-Ibrahim et al., 2022).

Previous studies have suggested that the recognition of functional actions follows a pathway from the occipital visual areas through the frontoparietal network to the ventral premotor areas, with the processing largely dependent on the visual dorso-ventral visual pathway (Augurelle et al., 2003). Furthermore, the critical role of the left frontoparietal network in functional action recognition has been highlighted (Buxbaum et al., 2006; Rueschemeyer et al., 2010; Salazar-López et al., 2016). In our study, we observed lateralized activation in the medial superior frontal gyrus during the 300–400 ms time window. Over time, the activation related to action recognition shifted from the occipital cortex (144–194 ms) to the frontoparietal network. At the later time window (562–576 ms), action recognition-related activation was observed not only in the frontal cortex but also in higher-level visual cognitive regions, such as the right superior occipital gyrus. These findings are consistent with prior research and underscore the importance of the occipital cortex in action recognition processing (Brandi et al., 2014).

### 4.2 Structural action semantic congruency-related effects

The semantic variable of structural actions was also manipulated in this experiment. Data analysis revealed neural responses related to structural actions during the 456–470 and 610–660 ms time windows. At 456–470 ms, significant activation was observed in the left postcentral gyrus and right middle temporal gyrus. At 610–660 ms, activation was concentrated in the bilateral precentral gyrus and widespread areas of the temporal lobe, including the left superior temporal gyrus and left middle temporal gyrus. These findings confirm the critical role of parietal regions in the recognition of structural actions (Augurelle et al., 2003; Karnath and Perenin, 2005; Perenin and Vighetto, 1988). Additionally, the significant activation observed in the temporal lobe further emphasizes the necessity of the temporal network within the visual ventral stream for processing action-related information (Al Harbi and Gotoh, 2015; Binkofski and Buxbaum, 2013; Grafton, 2010).

Furthermore, the observed time windows with differences in the semantics of structural actions correspond to those associated with the N400 component, which reflects semantic processing (Kutas and Federmeier, 2011; Leynes et al., 2024; Li and Wang, 2016), and the P600 component, which is typically linked to late-stage conflict resolution. The activation of both the N400 and P600 components predominantly occurs in the parietal lobe (Aguado et al., 2013) and the occipital-temporal cortex (Kim et al., 2024). These results provide strong evidence of significant differences in the semantic processing of structural actions.

### 4.3 Relationship between two types of manipulative actions

According to action recognition theory, the recognition of structural and functional actions generally follows distinct, independent visual pathways (Augurelle et al., 2003; Buxbaum and Kalenine, 2010). Experimental studies on action recognition have confirmed that different brain regions are activated during the recognition of these two actions (Buxbaum and Saffran, 2002; De Bellis et al., 2020; Karnath and Perenin, 2005; Rueschemeyer et al., 2010; Salazar-López et al., 2016). However, owing to differences in the content and attentional demands associated with processing the representations of these actions, some researchers have proposed that the recognition of structural actions forms the foundation for recognizing functional actions (Binkofski and Buxbaum, 2013; Buxbaum and Kalenine, 2010; Jax and Buxbaum, 2010). Additionally, the activation of certain occipital–parietal brain networks has been shown to overlap in the recognition of both action types (Cohen et al., 2009). Our results showed that structural actions semantic processing activates brain regions transitioning from the occipito-temporal to fronto-parietal areas, while functional actions recognition follows a pathway from the occipital lobe, through the parietal lobe, to the frontal cortex. This activation in the occipito-temporal region aligns with findings by Courson and Tremblay (2020) and Giacobbe et al. (2022). Furthermore, both action types activated motor regions in the fronto-parietal cortex, supporting the embodied cognition theory. The activation of the anterior and posterior central sulcus during structural actions is consistent with regions involved in hand movement (Klepp et al., 2014), highlighting the fronto-parietal cortex’s key role in action semantic processing.

In our study, we used action-related words as cues rather than simple gesture images to examine whether the semantic processing of structural actions influences the recognition of functional actions. Previous research has shown that understanding verbs and sentences related to bodily actions activates specific areas of the somatosensory and motor cortices, suggesting an advantage in action semantic processing (Liu et al., 2022; Monaco et al., 2023). By using action-related words as cues, we minimized the interference of image-based action information in recognition, thereby preserving the temporal sensitivity of the process. The finding that semantic congruence significantly reduced RTs supports this conclusion. However, the ERP results revealed that the semantic processing of structural actions and the recognition of functional actions are temporally independent, indicating that these two processes involve distinct neural mechanisms from the perspective of semantic processing. Additionally, this study has limitations, as it used a general university student population, whose cognitive abilities and action processing are superior to those of special populations, such as individuals with motor impairments. Future research could explore whether the recognition of manipulative actions in such populations is independent or involves interactions. Furthermore, differences in task settings may lead to distinct neural activations between the semantic processing of structural actions and their direct representations. Future research could compare the neural encoding of structural and functional actions or isolate structural (e.g., handle) and functional (e.g., hammerhead) object regions to provide more direct evidence for action recognition mechanisms. Manipulability enhances action recognition, as shown by studies where individuals with motor impairments performed better with manipulable objects (Beauprez et al., 2020). This aligns with embodied cognition theory, which links action cognition with body perception (Barsalou, 2008). Future research could use manipulable objects for memory and action cognition training in patients with motor impairments, such as Parkinson’s disease, using fMRI to observe brain activation and connectivity.

## 5 Conclusion

This study investigated whether the semantic cognitive processing of structural and functional actions is independent by manipulating different action types as stimuli. While semantic congruency in structural actions significantly reduced RTs for functional action judgments, neural activation patterns revealed independent processing. Significant differences in functional action recognition were observed in the left superior frontal gyrus (30–44 ms) and later in the frontal and occipital cortices (562–576 ms), with sustained activation from the occipital to parietal regions (144–194, 218–232, and 300–400 ms). In contrast, structural action-related differences appeared in the 456–470 and 610–660 ms time windows, with activation in the parietal and temporal regions linked to semantic processing and conflict resolution. These findings support the independence of structural and functional action semantic processing at the neural level. Future research should explore the implications of these independent pathways for rehabilitation and cognitive training in motor-impaired populations.

## Data Availability

The original contributions presented in the study are included in the article/supplementary material, further inquiries can be directed to the corresponding author.

## Footnotes

^1^ http://brainvisa.info

^2^ http://neuroimage.usc.edu/brainstorm
